# Supplementary material for: Prediction and Visualization of Non-Enhancing Tumor in Glioblastoma via T1w/T2w-Ratio Map
Source: Brain Sci. 2022 Jan 12;12(1):99. doi: 10.3390/brainsci12010099 (PMC8774070; doi:10.3390/brainsci12010099)
Supplement: Supplementary file 1 [file brainsci-12-00099-s001.zip › brainsci-1533070-supplementary/Supplementary/brain_sciences_supple.pdf]

brain sciences

Prediction and visualization of non-enhancing tumor in glioblastoma via T1w/T2w-ratio map

Shota Yamamoto [1,2], Takahiro Sanada [1], Mio Sakai [3], Atsuko Arisawa [4], Naoki Kagawa [2], Eku Shimosegawa [5], Katsuyuki Nakanishi [3], Yonehiro Kanemura [6], Manabu Kinoshita [1,2,7,*] ,Haruhiko Kishima [2]

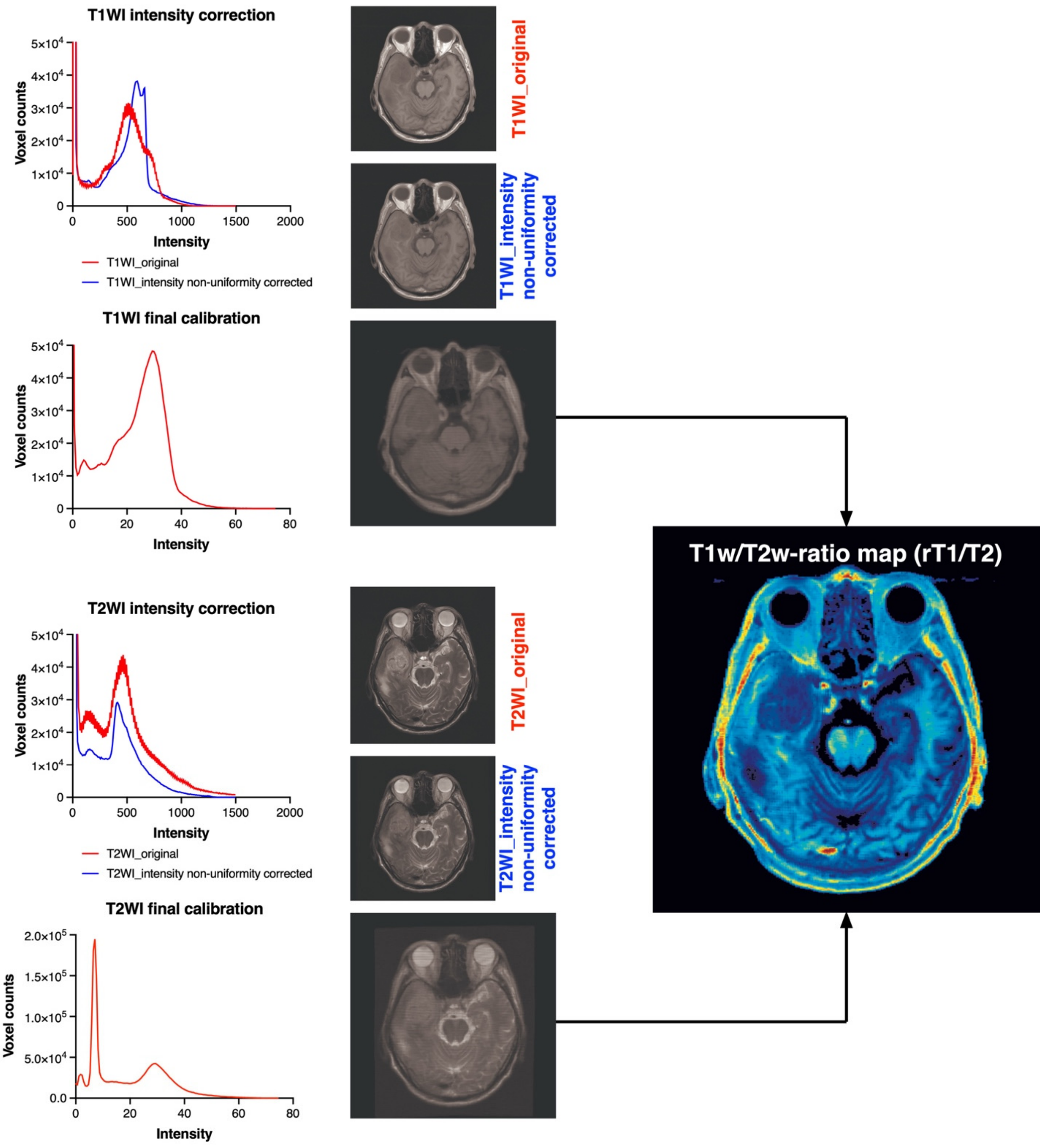

Figure S1. Image processing for T1w/T2w-ratio map (rT1/T2) reconstruction is presented. Both T1WI and T2WI were subjected to intensity non-uniformity correction and intensity normalization. T1w/T2w-ratio map (rT1/T2) was created by the finally calibrated and image intensity normalized T1- and T2WI. Further details can be found in the reference article 14 by Ganzetti et al [14].

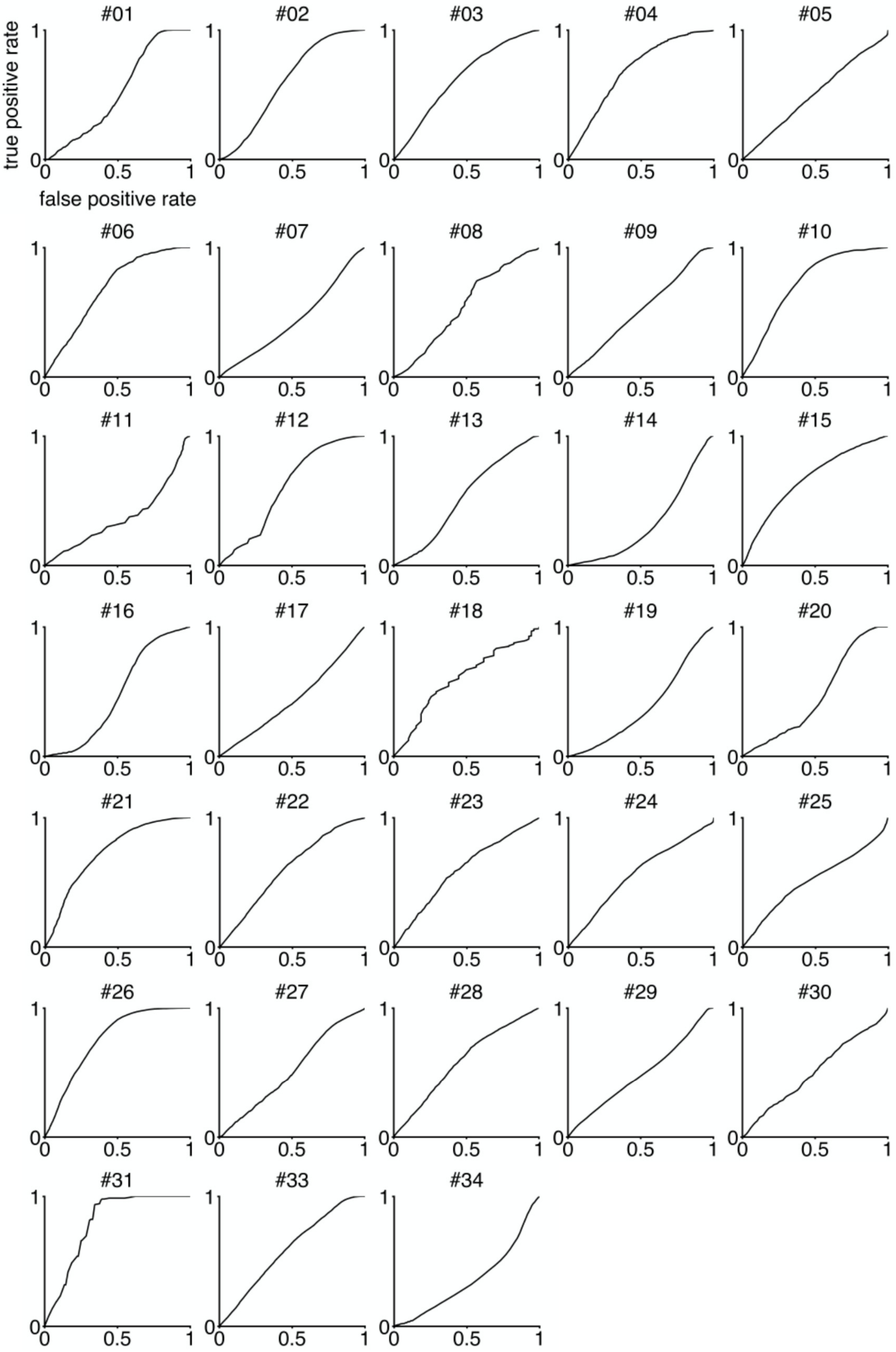

Figure S2. ROC curves of LMPH to detect Met-PET high for each case.

Table S1. Relationship between rT1/T2 and Likeliness of MET-PET high.

Numbers above black line indicate the range of rT1/T2 and below the line indicate the Likeliness of MET-PET high. Abbreviations: NaN, Not a number.

0.0-0.01	0.01-0.02	0.02-0.03	0.03-0.04	0.04-0.05	0.05-0.06	0.06-0.07	0.07-0.08	0.08-0.09	0.09-0.1
NaN	NaN	NaN	-1.000	-1.000	-1.000	NaN	-1.000	-1.000	-1.000

0.1-0.11	0.11-0.12	0.12-0.13	0.13-0.14	0.14-0.15	0.15-0.16	0.16-0.17	0.17-0.18	0.18-0.19	0.19-0.2
-1.000	-1.000	NaN	-1.000	-1.000	-1.000	-1.000	-1.000	-0.705	-1.000

0.2-0.21	0.21-0.22	0.22-0.23	0.23-0.24	0.24-0.25	0.25-0.26	0.26-0.27	0.27-0.28	0.28-0.29	0.29-0.3
-1.000	-0.770	-0.891	-0.908	-0.831	-0.936	-0.948	-0.872	-0.680	-0.643

0.3-0.31	0.31-0.32	0.32-0.33	0.33-0.34	0.34-0.35	0.35-0.36	0.36-0.37	0.37-0.38	0.38-0.39	0.39-0.4
-0.549	-0.705	-0.584	-0.662	-0.590	-0.564	-0.626	-0.632	-0.654	-0.688

0.4-0.41	0.41-0.42	0.42-0.43	0.43-0.44	0.44-0.45	0.45-0.46	0.46-0.47	0.47-0.48	0.48-0.49	0.49-0.5
-0.643	-0.655	-0.649	-0.689	-0.657	-0.621	-0.618	-0.584	-0.578	-0.510

0.5-0.51	0.51-0.52	0.52-0.53	0.53-0.54	0.54-0.55	0.55-0.56	0.56-0.57	0.57-0.58	0.58-0.59	0.59-0.6
-0.490	-0.429	-0.376	-0.354	-0.301	-0.271	-0.226	-0.216	-0.190	-0.162

0.6-0.61	0.61-0.62	0.62-0.63	0.63-0.64	0.64-0.65	0.65-0.66	0.66-0.67	0.67-0.68	0.68-0.69	0.69-0.7
-0.142	-0.120	-0.103	-0.105	-0.099	-0.087	-0.071	-0.068	-0.048	-0.019

0.7-0.71	0.71-0.72	0.72-0.73	0.73-0.74	0.74-0.75	0.75-0.76	0.76-0.77	0.77-0.78	0.78-0.79	0.79-0.8
-0.014	-0.004	0.016	0.015	0.029	0.026	0.040	0.031	0.035	0.028

0.8-0.81	0.81-0.82	0.82-0.83	0.83-0.84	0.84-0.85	0.85-0.86	0.86-0.87	0.87-0.88	0.88-0.89	0.89-0.9
0.041	0.041	0.049	0.067	0.086	0.094	0.112	0.129	0.145	0.163

0.9-0.91	0.91-0.92	0.92-0.93	0.93-0.94	0.94-0.95	0.95-0.96	0.96-0.97	0.97-0.98	0.98-0.99	0.99-1.0

0.179	0.183	0.176	0.165	0.158	0.160	0.144	0.128	0.121	0.108
1.0-1.01	1.01-1.02	1.02-1.03	1.03-1.04	1.04-1.05	1.05-1.06	1.06-1.07	1.07-1.08	1.08-1.09	1.09-1.1
0.102	0.103	0.124	0.143	0.123	0.160	0.161	0.174	0.140	0.113
1.1-1.11	1.11-1.12	1.12-1.13	1.13-1.14	1.14-1.15	1.15-1.16	1.16-1.17	1.17-1.18	1.18-1.19	1.19-1.2
0.124	0.143	0.102	0.139	0.152	0.122	0.088	0.102	0.072	0.019
1.2-1.21	1.21-1.22	1.22-1.23	1.23-1.24	1.24-1.25	1.25-1.26	1.26-1.27	1.27-1.28	1.28-1.29	1.29-1.3
0.014	0.044	0.095	0.183	0.109	0.119	0.166	0.206	0.221	0.119
1.3-1.31	1.31-1.32	1.32-1.33	1.33-1.34	1.34-1.35	1.35-1.36	1.36-1.37	1.37-1.38	1.38-1.39	1.39-1.4
0.109	0.062	0.139	0.116	0.199	0.146	0.201	0.181	0.185	0.177
1.4-1.41	1.41-1.42	1.42-1.43	1.43-1.44	1.44-1.45	1.45-1.46	1.46-1.47	1.47-1.48	1.48-1.49	1.49-1.5
0.249	0.237	0.397	0.004	0.218	0.256	0.249	0.243	0.382	0.218
1.5-1.51	1.51-1.52	1.52-1.53	1.53-1.54	1.54-1.55	1.55-1.56	1.56-1.57	1.57-1.58	1.58-1.59	1.59-1.6
0.470	0.871	0.218	0.019	-1.000	-0.486	-1.000	0.019	-0.092	-1.000
1.6-1.61	1.61-1.62	1.62-1.63	1.63-1.64	1.64-1.65	1.65-1.66	1.66-1.67	1.67-1.68	1.68-1.69	1.69-1.7
1.000	NaN	NaN	0.724	1.000	0.350	0.019	-1.000	NaN	1.000
1.7-1.71	1.71-1.72	1.72-1.73	1.73-1.74	1.74-1.75	1.75-1.76	1.76-1.77	1.77-1.78	1.78-1.79	1.79-1.8
1.000	1.000	NaN	1.000	1.000	1.000	NaN	NaN	NaN	1.000
1.8-1.81	1.81-1.82	1.82-1.83	1.83-1.84	1.84-1.85	1.85-1.86	1.86-1.87			
NaN	-1.000	-1.000	NaN	NaN	NaN	1.000			
